# Media optimization for economic succinic acid production by *Enterobacter* sp. LU1.

**DOI:** 10.1186/s13568-017-0423-0

**Published:** 2017-06-19

**Authors:** Marcin Podleśny, Agnieszka Kubik-Komar, Jagoda Kucharska, Jakub Wyrostek, Piotr Jarocki, Zdzisław Targoński

**Affiliations:** 1 Department of Biotechnology, Human Nutrition and Food Commodities, University of Life Sciences in Lublin, 8 Skromna, 20-704 Lublin, Poland; 2 Department of Applied Mathematics and Informatics, University of Life Sciences in Lublin, 28 Głęboka, 20-950 Lublin, Poland; 3 Department of Analysis and Food Quality Assessment, University of Life Sciences in Lublin, 8 Skromna, 20-704 Lublin, Poland

**Keywords:** *Enterobacter*, Succinic acid, Response surface methodology, Glycerol

## Abstract

*Enterobacter* sp. LU1 could efficiently convert glycerol to succinic acid under anaerobic conditions after the addition of lactose. In this study, media constituents affecting both *Enterobacter* sp. LU1 biomass and succinic acid production were investigated employing response surface methodology (RSM) with central composite design. Statistical methods led to the development of an efficient and inexpensive microbiological media based on crude glycerol, whey permeate as carbon sources and urea as a nitrogen source. The optimized production of bacterial biomass in aerobic conditions was predicted and the interactive effects between crude glycerol, urea and magnesium sulfate were investigated. As a result, a model for predicting the concentration of bacterial biocatalyst biomass was developed with crude glycerol as a sole carbon source. In addition, it was observed that the interactive effect between crude glycerol and urea was statistically significant. Response surface methodology was also employed to develop the model for predicting the concentration of succinic acid produced. Validity of the model was confirmed during verification experiments wherein actual results differed from predicted values by 0.77%. The applied statistical methods proved the feasibility for anaerobic succinic acid production on crude glycerol without expensive yeast extract addition. In conclusion, the RSM method can provide valuable information for succinic acid scale-up fermentation using *Enterobacter* sp. LU1.

**Electronic supplementary material:**

The online version of this article (doi:10.1186/s13568-017-0423-0) contains supplementary material, which is available to authorized users.

## Introduction

Succinic acid is one of the most promising substrates for the chemical industry. Noteworthy is that it is completely biodegradable and may be produced with environment-friendly technologies. For this reason, apart from traditional areas of application like the food, feed and pharmaceutical industries, it may be a potential constituent in the chemistry of polyesters (Delhomme et al. 2009). Although today succinic acid is produced mainly with chemical methods, a successive increase is observed in its biotechnological production (Reverdia, Myriant) wherein a key significance is ascribed to the applied biocatalyst (bacteria or yeast) (Jansen and Gulik 2014). In turn, effective biosynthesis with the use of these microorganisms requires appropriately selected production media. They are very often adjusted to specific metabolic requirements of the applied microorganisms, considering apart from taxonomic affiliation also their e.g. auxotrophic traits (McKinlay et al. 2010; Schröder et al. 2010). Importantly, it is recommended that the composition of culture medium be correlated with specific conditions of the process that also determine the growth requirements (Jansen et al. 2012). When considering industrial implementation of the biotechnological process, an equally important factor is the cost-effectiveness of its elements including, e.g., price and availability of substrates or certain chemical compounds constituting the microbiological culture medium or adjustment of their doses to be applied. Apart from the appropriate source of carbon, of the outmost significance is the choice of nitrogen source which in the case of complex organic substances like, e.g. yeast extract, despite the positive impact on succinic acid production process has also some drawbacks as it significantly increases fermentation costs (prices of the cheapest available extracts start from ca. 5 Euro/kg) and impairs the subsequent treatment processes (high load of organic matter which blocks, e.g., active surfaces of adsorbents and impairs filtration processes) (Lopez-Garzon and Straathof 2014). The possibility of producing succinic acid with the use of newly-isolated bacterial strain belonging to the genus *Enterobacter* has recently been described in literature (Podleśny et al. 2017). In a previous study, succinic acid was also produced by genetically modified *Enterobacter aerogenes* (Tajima et al. 2014). Recently proven succinic acid production capabilities are consistent with increasing load of data in scientific literature on biotechnological applications of *Enterobacter* genus. One of these is the ability of selected *Enterobacter* strains to produce ethanol onto glycerol or mannitol (Lee et al. 2012; Wang et al. 2013). From the viewpoint of biotechnology, significant is also the capability of producing hydrogen onto different waste materials (Mishra and Das 2013; Sarma et al. 2013). Apart from that, *Enterobacter* genus includes strains capable to degrade a wide spectrum of detrimental substances like for instance benzimidazole fungicides, trichloroethylene, glyphosate or MTBE (tert-butyl-methyl ether) (Barbera et al. 2011; Cycoń et al. 2011; Kang et al. 2012; Kryuchkova et al. 2014). In addition, bacteria of the genus *Enterobacter* may be applied in bioremediation processes to remove compounds of mercury, chromium or lead (Naik et al. 2012; Panda and Sarkar 2012; Sinha and Khare 2012). Of high market significance may also be the ability to produce enzymes used in various branches of the industry likes phytase (feed industry) or tannase (food, pharmaceutical, feed industries) (Singh et al. 2012; Yoon et al. 1996). Interesting seems also the positive effect of selected strains of the *Enterobacter* genus on the growth of crops expressed by their ability to bind atmospheric nitrogen, to absorb phosphorus compounds from soil and to produce 2,3-butanediol (D’Alessandro et al. 2013; Hardoim et al. 2013). Considering the metabolic predispositions of *Enterobacter* genus observed in existing studies, flexibility of its behaviors in varying environmental conditions, and possibility to apply similar metabolic engineering technology as for *Escherichia coli*, there is a great chance to include *Enterobacter* strains into the group of microorganisms with the highest biotechnological potential.

The objective of this study was, therefore, to develop microbiological culture medium, based on cheap and easily available substrates (waste glycerol and whey permeate), dedicated for the succinic acid production with *Enterobacter* sp. LU1. An attempt was also undertaken to select and optimize medium components for the proliferation process of *Enterobacter* sp. LU1 bacterial biomass on minimal culture media with the use of waste glycerol as sole carbon source. This approach would, simultaneously, allow introducing into practice a cheap culture medium for the propagation of the applied bacteria in order to prepare the inoculum for industrial-size bio-reactors.

## Materials and methods

### Microorganism

*Enterobacter* sp. LU1 was recently isolated from the goat rumen (Podleśny et al. 2017) and characterized as an efficient succinic acid producer on media containing glycerol and lactose. The *Enterobacter* sp. LU1 was deposited in International Culture Collection of Microorganisms at the Institute of Agricultural and Food Biotechnology under the number KKP 2050 (Warsaw, Poland). The strain was maintained frozen at −80 °C with 20% (w/w) glycerol added. Inoculum cultures were grown anaerobically at 37 °C in 100-mL serum bottles with a 50 mL working volume capped with gas-tight butyl rubber stoppers in the brain heart infusion medium (BHI) (Oxoid, UK), pH 7.4. Overnight cultures were used to inoculate the fermentation medium [10% (v/v)]. Prior to inoculation, the overnight cultures were centrifuged, and the bacterial biomass obtained was first suspended in a portion (5%) of the adequate tested microbiological medium and then combined with the rest.

### Optimization procedure

#### Plackett–Burman design

A Plackett–Burman design was employed to screen and identify significant media constituents for bacterial biomass and succinic acid production from waste glycerol by *Enterobacter* sp. LU1. The investigated medium ingredients were: crude glycerol (85.0%) (as a carbon source), urea, MgCO_3_; MgSO_4_ × 7H_2_O; CaCl_2_; K_2_HPO_4_; NaCl for both biomass production and succinic acid formation which was additionally tested for whey permeate. The Plackett-Burman design is based on the first order model (Eq. 1):

1 $$ Y = \beta_{0} + \sum {\beta_{i} X_{i} } $$

where: Y is the response (biomass or succinic acid concentration), β_0_ is the model intercept, β_i_ is the linear coefficient, and X_i_ is the level of the independent variable. Each of the variables in the Plackett–Burman design was examined at two levels: −1 for low level and +1 for high level. The factors significant at the 95% level (p ≤ 0.05) were considered to have a significant effect on biomass or succinic acid production by *Enterobacter* sp. LU1 and were further exploited in the CCD approach. Additional file 1: Table S1 and S3 show the high and low levels of each factor used in the Plackett–Burman design experiment.

#### CCD

Central composite design (CCD) was employed for the effect optimization of each significant variable on the responses, i.e., biomass and succinic acid concentration. The following quadratic model (Eq. 2) was used to optimize the key medium components (factors).

2 $$ Y = \beta_{0} + \sum {\beta_{i} X_{i} } + \sum {\beta_{i} X_{i}^{2} } + \sum {\beta_{j} X_{i} X_{j} } $$

where: Y is the predicted response (biomass and succinic acid concentration), β_0_ is the interception, β_i_ is the linear effect, β_ii_ is the quadratic effect, and β_ij_ are interaction effect coefficients. X_i_ and X_j_ are coded values of the factors selected as a result of the initial screening. The significance of the resulting model was checked by an F-test, and goodness of fit was tested by determining the R^2^ coefficient. The correlations between experimental and predicted values were shown on response surface plots. All designed matrices were generated and analyses were performed using Statistica software (version 8.0; StatSoft, Inc., http://www.statsoft.com). The media components of each trial are shown in Tables 2 and 4.

### Biomass and succinic acid production from waste glycerol

Biomass was produced in 100 mL Erlenmeyer flasks with a 20 mL working volume using waste glycerol as a substrate. Tests concerning succinic acid production were done in 100 mL serum bottles with a 20 mL working volume. The serum bottles were tightly sealed with rubber stoppers and aluminum caps [CO_2_ was the headspace gas in all serum bottle incubations (about 0.8 bar overpressure)]. All experiments were done at 34 °C for 22 h and 180 rpm when measuring biomass concentration and for 120 h and 160 rpm if succinic acid was produced. The concentration of each media component was adjusted according to design (Tables 1, 2, 3 and 4). All treatments were made in triplicate. Waste glycerol (85% v/w) used in the study originated from Czechowice-Dziedzice biodiesel production plant (LOTOS Group, Poland). Whey permeate powder was obtained from Spomlek Dairy Cooperative (Radzyn Podlaski, Poland) and it contained about 77% (v/w) of lactose.

**Table 1:** Plackett–Burman design for seven variables with the corresponding experimental values of biomass concentration

Run	Crude glycerol	Urea	MgCO_3_	MgSO_4_ × 7H_2_O	CaCl_2_	K_2_HPO_4_	NaCl	Biomass (g/L)
1	−1	−1	−1	+1	+1	+1	−1	1.10 ± 0.02
2	+1	−1	−1	−1	−1	+1	+1	1.48 ± 0.01
3	−1	+1	−1	−1	+1	−1	+1	1.32 ± 0.03
4	+1	+1	−1	+1	−1	−1	−1	4.63 ± 0.1
5	−1	−1	+1	+1	−1	−1	+1	1.08 ± 0.01
6	+1	−1	+1	−1	+1	−1	−1	1.45 ± 0.01
7	−1	+1	+1	−1	−1	+1	−1	1.42 ± 0.04
8	+1	+1	+1	+1	+1	+1	+1	8.03 ± 0.22

**Table 2:** Central composite design matrix of the independent variables with the corresponding experimental values of biomass concentration

Run	Crude glycerol (x_1_) (g/L)	Urea (x_2_) (g/L)	MgSO_4_ × 7H_2_O (x_3_) (g/L)	Biomass (g/L)
1	10	1	0.1	3.27 ± 0.02
2	30	1	0.1	6.99 ± 0.14
3	10	3	0.1	3.16 ± 0.24
4	30	3	0.1	7.89 ± 0.18
5	10	1	0.3	3.36 ± 0.04
6	30	1	0.3	6.83 ± 0.13
7	10	3	0.3	2.98 ± 0.15
8	30	3	0.3	7.47 ± 0.19
9	3.2	2	0.2	1.31 ± 0.03
10	36.8	2	0.2	8.08 ± 0.12
11	20	0.32	0.2	2.61 ± 0.09
12	20	3.68	0.2	5.26 ± 0.1
13	20	2	0.032	5.65 ± 0.15
14	20	2	0.368	6.24 ± 0.29
15	20	2	0.2	6.01 ± 0.12
16	20	2	0.2	5.94 ± 0.07
17	20	2	0.2	6.02 ± 0.11
18	20	2	0.2	6.01 ± 0.09
19	20	2	0.2	5.96 ± 0.15
20	20	2	0.2	6.01 ± 0.06

**Table 3:** Plackett–Burman design for eight variables with the corresponding experimental values of succinic acid concentration

Run	Crude glycerol	Whey permeate	Urea	MgCO_3_	K_2_HPO_4_	MgSO_4_ × 7H_2_O	CaCl_2_	NaCl	D1	D2	D3	Succinic acid (g/L)
1	+1	−1	−1	−1	+1	−1	+1	+1	−1	+1	+1	2.42 ± 0.22
2	−1	+1	−1	+1	+1	−1	+1	+1	+1	−1	−1	4.87 ± 0.67
3	−1	+1	+1	−1	+1	+1	+1	−1	−1	−1	+1	3.63 ± 0.13
4	−1	−1	−1	+1	−1	+1	+1	−1	+1	+1	+1	nd
5	+1	−1	+1	+1	+1	−1	−1	−1	+1	−1	+1	9.42 ± 0.15
6	−1	−1	+1	−1	+1	+1	−1	+1	+1	+1	−1	nd
7	+1	+1	−1	−1	−1	+1	−1	+1	+1	−1	+1	1.76 ± 0.34
8	−1	+1	+1	+1	−1	−1	−1	+1	−1	+1	+1	4.29 ± 0.12
9	+1	+1	+1	−1	−1	−1	+1	−1	+1	+1	−1	3.45 ± 0.16
10	+1	−1	+1	+1	−1	+1	+1	+1	−1	−1	−1	3.17 ± 0.45
11	+1	+1	−1	+1	+1	+1	−1	−1	−1	+1	−1	6.35 ± 0.02
12	−1	−1	−1	−1	−1	−1	−1	−1	−1	−1	−1	nd

**Table 4:** Central composite design matrix of the independent variables with the corresponding experimental values of succinic acid concentration

Run	Crude glycerol (x_1_)	MgCO_3_ (x_2_)	K_2_HPO_4_ (X_3_)	Succinic acid (g/L)
1	12.5	12.5	0.5	7.95 ± 0.08
2	27.5	12.5	0.5	14.2 ± 0.19
3	12.5	27.5	0.5	13.15 ± 0.03
4	27.5	27.5	0.5	20.27 ± 0.6
5	12.5	12.5	1.5	8.26 ± 0.13
6	27.5	12.5	1.5	14.2 ± 0.02
7	12.5	27.5	1.5	13.29 ± 0.6
8	27.5	27.5	1.5	19.75 ± 0.45
9	7.4	20	1	10.13 ± 0.01
10	32.6	20	1	17.62 ± 0.67
11	20	7.4	1	9.6 ± 0.2
12	20	32.6	1	16.09 ± 0.45
13	20	20	0.16	15.42 ± 0.24
14	20	20	1.84	16.08 ± 0.56
15	20	20	1	16.38 ± 0.13
16	20	20	1	16.19 ± 0.38
17	20	20	1	16.68 ± 0.28
18	20	20	1	16.29 ± 0.09
19	20	20	1	15.98 ± 0.19
20	20	20	1	15.99 ± 0.33

### Analytical methods

Cell growth was monitored by measuring the absorbance of the broth at 600 nm (OD600) after diluting the sample 1:1 with 7% HCl (v/v). The biomass concentration of the *Enterobacter* sp. LU1 strain was estimated by determining the dry cell weight (DCW) using a predetermined correlation curve obtained between the absorbance measured at 600 nm and the cell dry weight (g/l). One unit of OD600 was roughly equivalent to 0.51 g/l of DCW for cells of *Enterobacter* sp. LU1 grown in BHI medium (Podleśny et al. 2017). Samples for succinic acid and by-product detection were prepared by centrifugation of the culture broth at 6000×*g* for 5 min. The resulting supernatant, after dilution with water (1:1), was analyzed by high-performance liquid chromatography system (Gilson) equipped with an ion exchange column (Aminex HPX-87H, BioRad) and a refractive index detector using 0.03 M sulfuric acid as mobile phase at 42 °C (Dharmadi et al. 2006).

## Results

### Statistical optimization of medium composition for *Enterobacter* sp. LU1 cultivation

Waste glycerol, urea, MgCO_3_, MgSO_4_ × 7H_2_O, CaCl_2_, K_2_HPO_4_ and NaCl were used to compose the Plackett–Burman design (Additional file 1: Table S1). Effects of the variables on the response and the estimated values for the effect of each of the independent factors are shown in Table 1. The highest *Enterobacter* sp. LU1 biomass concentration (8.03 g/L) was observed in experiment 8, in which all the variables were at their highest levels. All studied factors exerted a positive effect on biomass production. Nonetheless waste glycerol, urea and magnesium sulfate concentration had the strongest impact on the level of *Enterobacter* sp. LU1 biomass concentration. Therefore, these three factors were chosen for further study (Additional file 1: Figure S1). Based on the screening of variables by 2-level Plackett–Burman design, full-factorial central composite design (CCD) was developed for variables significantly affecting biomass production and consisted of five levels: the low and high levels (−1 and +1), central points (0) and star points with α ± 1.68, giving twenty combinations of cultivation parameters as shown in Table 2. Other factors tested previously were set at their high levels according to their positive effects (MgCO_3_ 3 g/L, CaCl_2_ 0.5 g/L, K_2_HPO_4_ 1 g/L and NaCl 1 g/L). As it can be seen in Table 2, the highest concentration of biomass was obtained in run no. 10 (8.08 g/L) where concentrations of crude glycerol, urea and magnesium sulfate in the medium were as follows g/L: 36.8; 2.0 and 0.2. In turn, minimal biomass concentration was obtained in run no. 9 (1.31 g/L) where we could find 3.2 g/L of crude glycerol, 2 g/L of urea and 0.2 g/L of magnesium sulfate. On the basis of the CCD analysis and regression coefficients obtained (Additional file 1: Table S2) following simplified quadratic equation was calculated (Eq. 3):

3 $$ Y = 6.0406 + 2.0360 \, X_{1} - 0.3687 \, X_{1}^{2} + 0.4017 \, X_{2} - 0.6407 \, X_{2}^{2} + 0.2549 \, X_{1} X_{2} $$

**Fig. 1:**
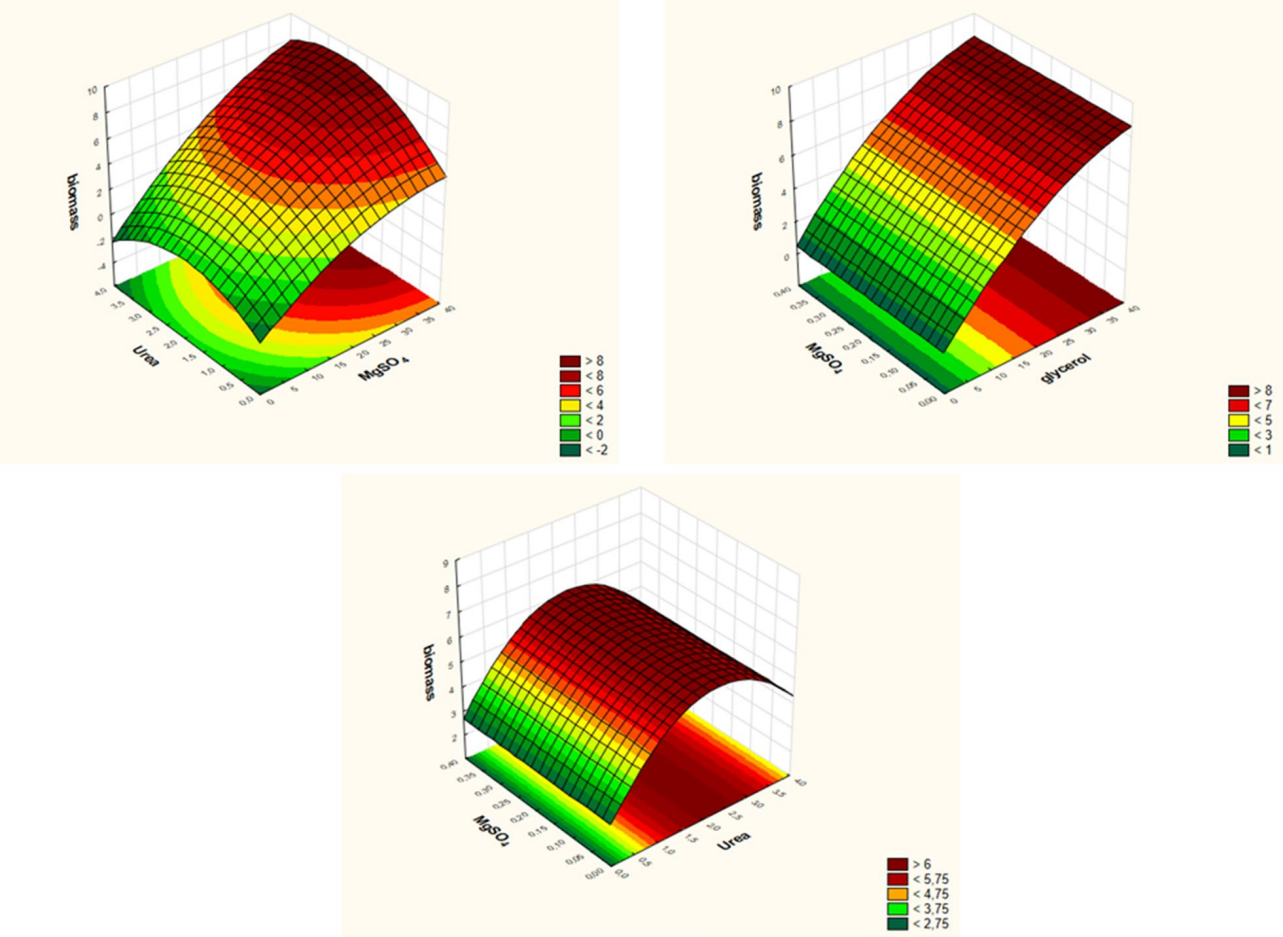
Response surface plots showing the effects of (x_1_) crude glycerol, (x_2_) urea and (x_3_) magnesium sulfate on (Y) biomass concentration (g/L)

The calculated R^2^ value of 0.95993 suggests that this model is well fitted to the experimental data. The response surface plots are shown in Fig. 1. These plots demonstrate that biomass concentration was affected by 2 of the 3 investigated factors namely crude glycerol and urea. In the case of magnesium sulfate, no influence was observed in the studied range of its concentrations (0.032–0.368 g/L). The selected range covered the optimum condition for urea and for the crude glycerol respond to suboptimal conditions. However, chromatographic analysis showed incomplete consumption of 36.8 g/L glycerol in the medium which may suggest that because of its “crude” nature it contains some other non-monitored micronutrients that also affect biomass production. Additionally, the obtained results indicate a minimal potential increase of biomass concentration upon further glycerol concentration rise, and therefore could be economically inefficient. Optimum biomass production by *Enterobacter* sp. LU1 as defined on the basis of RSM occurs using: concentration of glycerol 36.8 g/L, urea 2.84 g/L and magnesium sulfate 0.2 g/L. Concentrations of other medium constituents were at the same level as previously. In such conditions, the predicted concentration of biomass after 22 h of incubation using the mathematical model is 8.6653 g/L. In order to validate the adequacy of the model, the confirmation experiment was carried out using medium prepared according to the RSM results (the remaining medium components at the following concentrations g/L: MgCO_3_ 3.0, CaCl_2_ 0.5, K_2_HPO_4_ 1.0 and NaCl 1.0). The mean concentration of the obtained biomass from triplicate 22 h trials in a shaking flask was 8.7342 ± 0.38 which is about 0.8% higher than the predicted value and confirms the validity of the model.

### Statistical optimization of medium composition for succinic acid production by *Enterobacter* sp. LU1

The Plackett–Burman design was also employed to evaluate the influence of culture medium components on succinic acid production by *Enterobacter* sp. LU1 using waste glycerol and whey permeate as carbon sources. Design for 12 trials with two levels of concentrations for each variable was exploited and consisted of eight medium components and three dummy variables/unassigned variables. Results of the experiments performed on the basis of the Plackett–Burman design are presented in Table 3. The highest production of succinic acid was observed in experiment 5 (9.42 g/L) and the levels of medium components that provided the highest response are indicated in Additional file 1: Table S3. Except calcium chloride, all tested medium components affected succinic acid production (response). A positive effect was observed with MgCO_3_, K_2_HPO_4_, waste glycerol, urea and whey permeate (Additional file 1: Figure S2). In contrast, magnesium sulfate and sodium chloride exerted a negative influence. Magnesium carbonate, dipotassium phosphate and waste glycerol were selected as the factors for CCD. Succinic acid concentrations for each individual run are summarized in Table 4. The highest succinic acid concentration of 20.27 g/L was obtained when the concentrations of magnesium carbonate, dipotassium phosphate and waste glycerol were 27.5, 0.5, and 27.5 g/L, respectively (Run 4). Also the lowest succinic acid concentration was 7.95 g/L, which was achieved when magnesium carbonate, dipotassium phosphate and waste glycerol concentrations were 12.5, 0.5, and 12.5 g/L, respectively (Table 4). On the basis of the CCD analysis and regression coefficients obtained (Additional file 1: Table S4) following simplified quadratic equation was calculated (Eq. 4):

4 $$ Y_{SA} = 16.0879 + 2.8097 \, X_{1} {-}0.8531 \, X_{1}^{2} + 2.3992 \, X_{2} {-}1.2162 \, X_{2}^{2} + 0.1743 \, X_{1} X_{2} $$

**Fig. 2:**
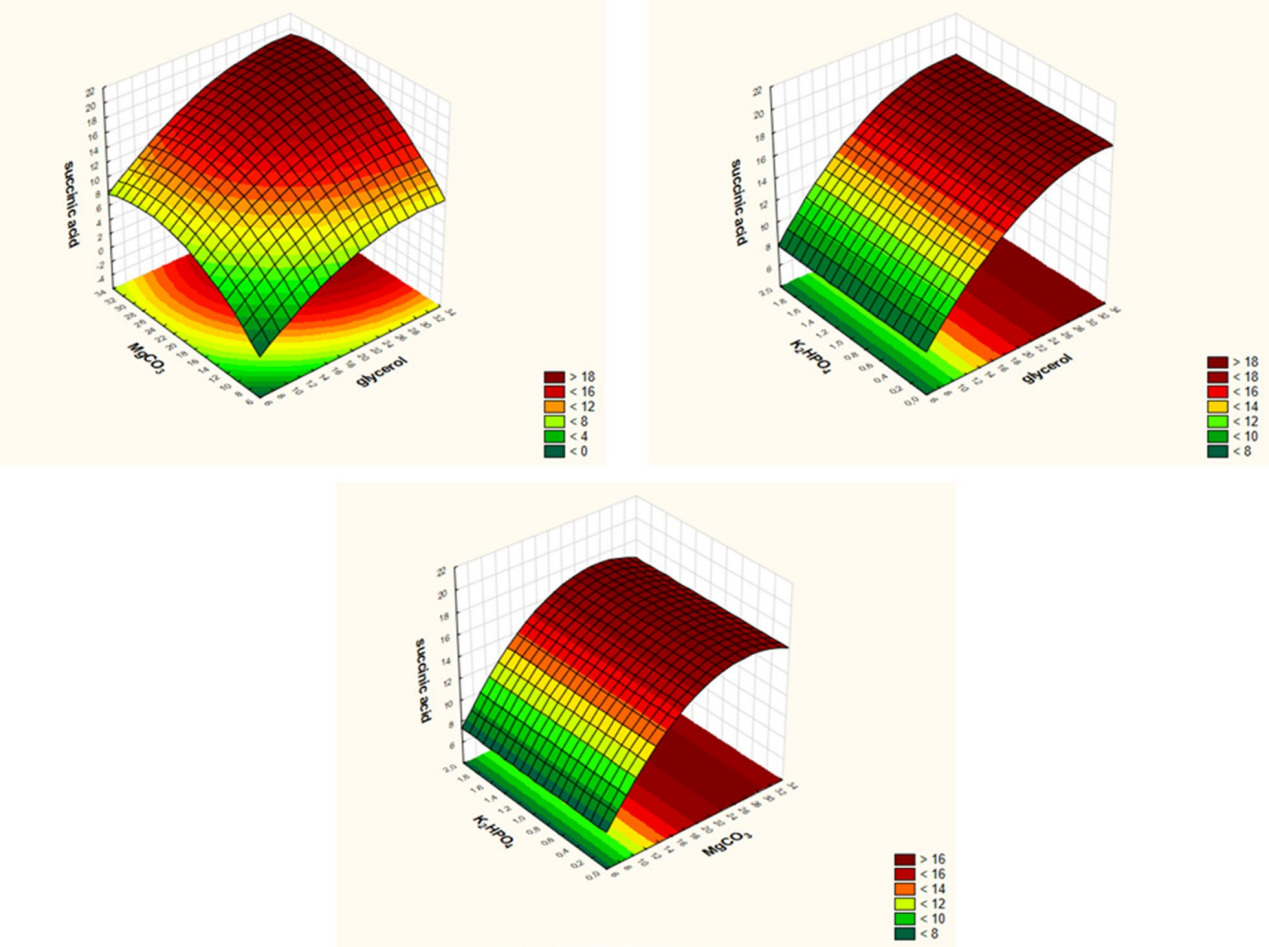
Response surface plots showing the effects of (x_1_) crude glycerol, (x_2_) magnesium carbonate and (x_3_) dipotassium phosphate on (Y) succinic acid concentration (g/L)

The fit of the model was examined by the coefficient of determination *R* ^*2*^, which was calculated to be 0.95774. This indicates that the sample variation of more than 95.8% was attributed to the given independent variable. The value of the adjustment determination coefficient (Adj *R* ^*2*^ = 95.38%) was also high to confirm the significance of the model. The three-dimensional graph for the response surface model is shown in Fig. 2. It is evident from the plot that succinic acid production by *Enterobacter* sp. LU1 was affected by crude glycerol and magnesium carbonate concentration and the influence of dipotassium phosphate was not confirmed within the tested concentration range. When the magnesium carbonate was at a low level, an increase in crude glycerol did not improve succinic acid concentration. However, if the crude glycerol and magnesium carbonate were at high levels, more succinic acid could be obtained. For magnesium carbonate the selected range covered the optimum condition but for crude glycerol it was suboptimal. Despite the last, similar to biomass optimization experiments, the amount of glycerol present in the medium was not completely consumed and as shown on surface plot further glycerol concentration increase could give only faintly improvement in succinic acid concentration. At least, it could be economically unjustified. Optimal succinic acid production by *Enterobacter* sp. LU1 as defined on the basis of RSM occurs using: glycerol in the concentration 32.6 g/L, MgCO_3_ 26.3 g/L and K_2_HPO_4_ 1.0 g/L. Other medium constituents include whey permeate (20 g/L) and urea (2 g/L). In such conditions, the predicted concentration of succinic acid after 120 h of incubation using the mathematical model is 19.804 g/L. In order to validate the adequacy of the model, the confirmation experiment was carried out using medium prepared according to the RSM results. The mean concentration of succinic acid from triplicate 120 h trials in serum bottles was 19.957 ± 0.22 g/L which is about 0.77% higher than the predicted value and confirms the validity of the model.

## Discussion

In works published so far on the microbiological production of succinic acid, bacteria applied have been usually characterized by high nutritive requirements in terms of fermentation media (necessity of using expensive organic supplements like e.g. yeast extract or peptone), just to mention *Actinobacillus succinogenes* (Guettler et al. 1999) or *Basfia succiniciproducens* (Schröder et al. 2010). Only part of the genetically-modified strains of *E. coli* (e.g. KJ060 or KJ134) were capable of succinic acid production on poor microbiological culture media (Jantama et al. 2008). Relatively sparse works have also been devoted to the use of waste glycerol as the basic substrate of this process moreover, conducted usually with the addition of organic extracts (Scholten et al. 2009; Vlysidis et al. 2011). Considering the above, increasing interest has been observed in the studies aimed at developing economic culture media for the microbiological production of succinic acid, particularly these recommending waste glycerol in medium composition. Earlier obtained results regarding succinic acid production with the use of *Enterobacter* sp. LU1 demonstrated a significant potential of this acid production on culture media with glycerol and lactose (Podleśny et al. 2017). Unfortunately, the achieved succinic acid concentration of 37 g/L required yeast extract addition in a dose as high as 15 g/L. The industrial implementation of this process would also require development of a microbiological culture medium based on cheap and easily-available substrates. The previously applied pure glycerol was replaced by waste glycerol from a bio-refinery that produces fatty acid methyl esters, lactose was replaced by whey permeate, whereas urea was used instead of the yeast extract as a source of nitrogen. Urea appears relatively often in works addressing the optimization and production of various bio-products on impoverished microbiological media (Wang et al. 2012). The choice of urea was also supported by the fact that due to its molecular composition—the supply of ammonium ions as a nitrogen source in the process additionally results in the release of carbon dioxide which is a significant substrate for carboxylic enzymes in the succinic acid production pathway (Tajima et al. 2015). The possibility of accelerating the succinic acid production process by introducing aeration at the early stage of cells proliferation as well as no need for lactose addition at this stage have prompted the individual approach to each stage of the process. The present study demonstrates the feasibility of substituting pure glycerol with waste glycerol and indicates whey permeate to be the appropriate source of lactose. Furthermore, optimization experiments confirmed the possibility of producing bacterial biomass of *Enterobacter* sp. LU1 on a simple culture medium with waste glycerol but without lactose addition (in any form). Results achieved in this study confirm also the feasibility of replacing expensive organic extracts with significantly cheaper urea. Although the first results achieved for *Enterobacter* sp. LU1 in succinic acid production without organic sources of nitrogen demonstrated a significant decrease in acid concentration in the medium, they were obtained during analyses made in small volumes (8 mL), corresponding to almost half of the total volume of the culture vessel (20 mL) (Podleśny et al. 2017). In optimization experiments, however, use was made of larger vessels (100-mL bottles) and the medium constituted not more than 20% of the total volume. In addition, the described microorganism cultured on glycerol was characterized by enhanced gas production in the samples based exclusively on simple sources of nitrogen, compared to YE. This observation has as yet not been elucidated, but knowing that hydrogen is one of the main gaseous components—its negative effect on *E. coli* capability for glycerol consumption in the fermentation process is expected (Dharmadi et al. 2006). In addition, considering the fact that the above-mentioned process of fermentative consumption of glycerol by *E. coli* is linked with the activity of formate-hydrogen lyase enzyme, the increased volume of the culture vessel may also be significant (Murarka et al. 2008) and may—in part—explain the results achieved earlier with *Enterobacter* sp. LU1 (Podleśny et al. 2017). The following medium components were analyzed apart from urea in optimization experiments: MgCO_3_; K_2_HPO_4_; MgSO_4_; CaCl_2_ and NaCl. The use of waste glycerol or—as in the case of culture medium for succinic acid production—whey permeate enabled omitting analyses of microelements that originated from these components (Pescuma et al. 2015; Samul et al. 2014). In the case of bacterial biomass production, all analyzed components of the culture medium had a positive effect upon this process. The greatest impact was observed in the case of the addition of glycerol, urea and magnesium sulfate. The organic source of carbon—herein waste glycerol—is a key growth-enhancing factor for heterotrophic microorganisms which additionally stimulates their metabolic functions. Another significant factor turned out to be magnesium sulfate comprising Mg^2+^ ions and sulfur. Sulfur is an inseparable element of some peptide chains as well as compounds being significant in the course of energetic processes in a cell (Sękowska et al. 2000). In turn, magnesium is not only a divalent ion significant for the functioning of many enzymes but also a key element of the structure of ribosomes or cell membranes (Maguire and Cowan 2002). Other factors, like MgCO_3_, K_2_HPO_4_, CaCl_2_ and NaCl had also a positive but less significant effect upon the process described. The above observations affected further optimization works aimed at establishing the most optimal for *Enterobacter* sp. LU1 bacterial biomass production concentrations of medium components having the strongest impact on this process. The application of central composite design allowed minimizing the number of necessary experiments in order to achieve statistically significant results. The conducted experiments confirmed the significance of two out of three analyzed at this stage factors, i.e. glycerol and urea, to the investigated process. The effect of the third one (MgSO_4_ × 7H_2_O) was found statistically insignificant in the analyzed range of concentrations. Perhaps, its effect could be noticeable only when coupled with the other components of the culture medium which at the first stage also exerted a significant effect on the process. Secondly, considering such a strong effect of glycerol and urea determined in the study, establishing the effect of magnesium sulfate may be impaired. Nonetheless, results achieved enabled creating a mathematical model which appeared very well fitted to the analyzed process and during verification with experimental data it provided results differing from the experimental ones by only 0.8%. Finally, we managed to establish optimal composition of microbiological culture medium for the quantity of bacterial biomass produced. It assumes the addition of the following components (g/L): glycerol 36.8, urea 2.84, magnesium sulfate 0.2, MgCO_3_ 3.0, CaCl_2_ 0.5, K_2_HPO_4_ 1.0, and NaCl 1.0. Summarizing, the culture medium developed assumes the addition of a cheap and easily available source of carbon—namely waste glycerol, and in the case of nitrogen source it is based on a relatively cheap urea. The remaining components of the medium are also strongly competitive in terms of price but, ultimately, make the described culture medium a perfect alternative for standard media applied in enterobacteria cultures like e.g. Luria–Bertani broth (Sezenov et al. 2007).

The approach to the choice of medium components for the stage of succinic acid product was analogous to that applied in the case of bacterial biomass production. Firstly, analyses were aimed at confirming the necessity of addition of its individual components. An additional element checked in respect of succinic acid production under anaerobic conditions was the addition of whey permeate containing mainly lactose (77%). Experiments carried out based on the Plackett–Burman design demonstrated the advisability of adding the following components to the microbiological medium: MgCO_3_, K_2_HPO_4_, glycerol, urea and whey permeate. In turn, the addition of MgSO_4_, NaCl and CaCl_2_ would have a negative effect on succinic acid production by *Enterobacter* sp. LU1. In addition, the impact of CaCl_2_ turned out statistically insignificant. The key importance of MgCO_3_ for the production of succinic acid by bacteria is relatively well document in different research works (Lee et al. 1999; Zhang et al. 2012). This compound is responsible for the control of medium pH and delivers Mg^2+^ ions being indispensable for the key enzyme in the succinic acid biosynthesis pathway, namely phosphoenolpyruvate carboxykinase. Based on results achieved, three components having the strongest impact on the investigated process were selected for further studies. Using the central composite design, a series of experiments were planned differing in the applied concentrations of MgCO_3_, K_2_HPO_4_ and glycerol. The analysis of results achieved in these experiments confirmed the effect of MgCO_3_ and glycerol on the process of succinic acid production by *Enterobacter* sp. LU1. Obviously, the significant impact of the source of carbon on succinic acid synthesis is not a surprise. Most of works devoted to the optimization of fermentation medium composition emphasize the importance of the choice of carbon source (Isar et al. 2006; Ji et al. 2009). In the case of the third factor, i.e. K_2_HPO_4_, no statistical significance of this variable was observed in the analyzed CCD model. A similar observation was made when optimizing the composition of the culture medium for succinic acid production with the use of *Corynebacterium glutamicum* (Jeon et al. 2013). Here also the application of Plackett–Burman design resulted in K_2_HPO_4_ inclusion among factors significant for the production of succinic acid, whereas at the successive stage of optimization involving the choice of appropriate concentrations of the selected factors, the effect of potassium phosphate turned out minimal. Nevertheless, based on the achieved mathematical model, culture medium composition was developed that confirmed the usability of this model for description of the analyzed process of succinic acid production in relation to the composition of the applied fermentation medium. Results of the verification experiment predicted with the same model diverged only by 0.77% from the experimental results, which allows concluding that the developed model fitted well to the real biotechnological process. The optimal for succinic acid synthesis concentrations of the applied components were as follows (g/L): MgCO_3_ 26.3, glycerol 32.6, K_2_HPO_4_ 1.0, whey permeate 20.0, and urea 2.0. In this way, results achieved confirmed the feasibility of conducting the process of succinic acid production on the culture medium not containing yeast extract or peptones, which enables significant reduction of costs of preparing such a culture medium and bring the described process of succinic acid production by *Enterobacter* sp. LU1 closer to the economic profitability.

## Additional file

**Additional file 1: Table S1.** Experimental ranges and levels of the 7 factors tested in the Plackett-Burman design for biomass production. **Table S2.** Regression coefficients and tests of their statistical significance for the simplified quadratic model for biomass production. **Table S3.** Experimental ranges and levels of the 8 factors tested in the Plackett-Burman design for succinic acid production. **Table S4.** Regression coefficients and tests of their statistical significance for the simplified quadratic model for succinic acid production. **Figure S1.** Pareto chart ranking the variables investigated in the Plackett-Burman design for biomass production. **Figure S2.** Pareto chart ranking the variables investigated in the Plackett-Burman design for succinic acid production.

## Abbreviations

BHI: brain heart infusion broth
CCD: central composite design
OD600: optical density at 600 nm
RSM: response surface methodology
YE: yeast extract
