# Supplementary material for: Media optimization for economic succinic acid production by Enterobacter sp. LU1
Source: AMB Express. 2017 Jun 19;7:126. doi: 10.1186/s13568-017-0423-0 (PMC5476557; doi:10.1186/s13568-017-0423-0)
Supplement: Supplementary file 1 — Additional file 1: Table S1. Experimental ranges and levels of the 7 factors tested in the Plackett-Burman design for biomass production. Table S2. Regression coefficients and tests of their statistical significance for the simplified quadratic model for biomass production. Table S3. Experimental ranges and levels of the 8 factors tested in the Plackett-Burman design for succinic acid production. Table S4. Regression coefficients and tests of their statistical significance for the simplified quadratic model for succinic acid production. Figure S1. Pareto chart ranking the variables investigated in the Plackett-Burman design for biomass production. Figure S2. Pareto chart ranking the variables investigated in the Plackett-Burman design for succinic acid production. [file 13568_2017_423_MOESM1_ESM.docx]

**Supplementary material.**

**Media optimization for economic succinic acid production by *Enterobacter* sp. LU1.**

Marcin Podleśny*^1^, Agnieszka Kubik-Komar^2^, Jagoda Kucharska^1^, Jakub Wyrostek^3^, Piotr Jarocki^1^, Zdzisław Targoński^1^

^1^Department of Biotechnology, Human Nutrition and Food Commodities, University of Life Sciences in Lublin, 8 Skromna, 20-704 Lublin, Poland

^2^Department of Applied Mathematics and Informatics, University of Life Sciences in Lublin, Akademicka 13, 20-950 Lublin, Poland

^3^Department of Analysis and Food Quality Assessment, University of Life Sciences in Lublin, 8 Skromna, 20-704 Lublin, Poland

*Corresponding author: Marcin Podleśny

E-mail:[podlesnymarcin@hotmail.com](mailto:podlesnymarcin@hotmail.com);

**Table S1** Experimental ranges and levels of the 7 factors tested in the Plackett-Burman design for biomass production.

| Independent variables | Code levels  -1 1 | |
| --- | --- | --- |
| Crude glycerol (g/L) | 2 | 20 |
| Urea (g/L) | 0.1 | 1 |
| MgCO_3_ (g/L) | 0.8 | 3 |
| MgSO_4_ x 7H_2_O (g/L) | 0.1 | 0.5 |
| CaCl_2_ (g/L) | 0 | 0.5 |
| K_2_HPO_4_ (g/L) | 0.1 | 1 |
| NaCl (g/L) | 0 | 1 |

**Table S2** Regression coefficients and tests of their statistical significance for the simplified quadratic model for biomass production.

|  | Regression coefficient | Stdandard Error | t(74) | p |
| --- | --- | --- | --- | --- |
| Mean/Intercept | 6.0406 | 0.06715 | 89.9573 | 0.000000 |
| X_1_ | 2.0360 | 0.052495 | 38.7839 | 0.000000 |
| X_1_ ^2^ | -0.3687 | 0.050913 | -7.2415 | 0.000000 |
| X_2_ | 0.4017 | 0.052495 | 7.6526 | 0.000000 |
| X_2_ ^2^ | -0.6407 | 0.050913 | -12.584 | 0.000000 |
| X_1_ X_2_ | 0.2549 | 0.068558 | 3.7184 | 0.000387 |

**Table S3** Experimental ranges and levels of the 8 factors tested in the Plackett-Burman design for succinic acid production.

| Independent variables | Code levels  -1 1 | |
| --- | --- | --- |
| Crude glycerol (g/L) | 1 | 30 |
| Whey permeate (g/L) | 5 | 20 |
| Urea (g/L) | 0.5 | 2 |
| MgCO_3_ (g/L) | 2 | 20 |
| K_2_HPO_4_ (g/L) | 0 | 1 |
| MgSO_4_ x 7H_2_O (g/L) | 0 | 1 |
| CaCl_2_ (g/L) | 0 | 1 |
| NaCl (g/L) | 0 | 1 |

**Table S4** Regression coefficients and tests of their statistical significance for the simplified quadratic model for succinic acid production.

|  | Regression coefficient | Standard Error | t(54) | p |
| --- | --- | --- | --- | --- |
| Mean/Intercept | 16.0879 | 0.145507 | 110.5643 | 0.000000 |
| X_1_ | 2.8097 | 0.113751 | 24.7007 | 0.000000 |
| X_1_ ^2^ | -0.8531 | 0.110324 | -7.7329 | 0.000000 |
| X_2_ | 2.3992 | 0.113751 | 21.0915 | 0.000000 |
| X_2_ ^2^ | -1.2162 | 0.110324 | -11.0241 | 0.000000 |
| X_1_ X_2_ | 0.1743 | 0.148557 | 1.1732 | 0.245847 |

**Figure S1.** Pareto chart ranking the variables investigated in the Plackett-Burman design for biomass production.

**Figure S2.** Pareto chart ranking the variables investigated in the Plackett-Burman design for succinic acid production.
